# Executive Control of Attention in Narcolepsy

**DOI:** 10.1371/journal.pone.0033525

**Published:** 2012-04-25

**Authors:** Sophie Bayard, Muriel Croisier Langenier, Valérie Cochen De Cock, Sabine Scholz, Yves Dauvilliers

**Affiliations:** 1 National Reference Network for Narcolepsy, Department of Neurology, Hôpital Gui-de-Chauliac, CHU Montpellier, Montpellier, France; 2 Inserm U1061, University of Montpellier 1, Montpellier, France; The Chinese University of Hong Kong, Hong Kong

## Abstract

**Background:**

Narcolepsy with cataplexy (NC) is a disabling sleep disorder characterized by early loss of hypocretin neurons that project to areas involved in the attention network. We characterized the executive control of attention in drug-free patients with NC to determine whether the executive deficits observed in patients with NC are specific to the disease itself or whether they reflect performance changes due to the severity of excessive daytime sleepiness.

**Methodology:**

Twenty-two patients with NC compared to 22 patients with narcolepsy without cataplexy (NwC) matched for age, gender, intellectual level, objective daytime sleepiness and number of sleep onset REM periods (SOREMPs) were studied. Thirty-two matched healthy controls were included. All participants underwent a standardized interview, completed questionnaires, and neuropsychological tests. All patients underwent a polysomnography followed by multiple sleep latency tests (MSLT), with neuropsychological evaluation performed the same day between MSLT sessions.

**Principal Findings:**

Irrespective of diagnosis, patients reported higher self-reported attentional complaints associated with the intensity of depressive symptoms. Patients with NC performed slower and more variably on simple reaction time tasks than patients with NwC, who did not differ from controls. Patients with NC and NwC generally performed slower, reacted more variably, and made more errors than controls on executive functioning tests. Individual profile analyses showed a clear heterogeneity of the severity of executive deficit. This severity was related to objective sleepiness, higher number of SOREMPs on the MSLT, and lower intelligence quotient. The nature and severity of the executive deficits were unrelated to NC and NwC diagnosis.

**Conclusions:**

We demonstrated that drug-free patients with NC and NwC complained of attention deficit, with altered executive control of attention being explained by the severity of objective sleepiness and global intellectual level. Further studies are needed to explore whether medications that promote wakefulness can improve the executive functions in narcolepsy.

## Introduction

Narcolepsy with cataplexy (NC) is a rare disabling disorder characterized by excessive daytime sleepiness (EDS), cataplexy, and other dissociated manifestations of rapid eye movement (REM) sleep phenomena such as sleep paralysis and hypnagogic hallucinations [1]. Narcolepsy was further classified into narcolepsy with and without (NwC) cataplexy (ICSD2). Recent pathophysiological studies have demonstrated that NC is caused by the early loss of hypothalamus neurons that produce hypocretin/orexin, a wakefulness-associated neurotransmitter that can be measured in the cerebrospinal fluid (CSF) [1]–[4]. In contrast, NwC is rarely caused by hypocretin deficiency, and affects only 10–30% of patients, supporting the concept of different pathogeneses in central hypersomnias [1]–[4].

NC significantly interferes with several aspects of daily life, wielding negative social and professional impacts that may considerably affect the quality of life [5]–[9]. Hence, patients with NC frequently have problems at school and at home, and find it difficult to obtain and maintain employment and to form relationships [10], [11]. Most of these behavioural difficulties may be attributable to the integrity of the executive functions, including distractibility, decision-making and organization problems, and difficulties in making, carrying out, and adjusting plans [12]. Recent studies have reported clear interactions between EDS and executive functioning [13]. Hypocretin has been reported as an essential neural substratum for many types of motivated behaviours [14]. To date, it remains unclear whether the limitations noted in patients with NC are related to the severity of EDS or are specific to NC, i.e., due to hypocretin deficiency.

Studies on cognitive functioning in central hypersomnia are rare. They have generally addressed memory, attention areas and decision-making, focusing on NC only [15]–[18]. Early findings showed that patients with NC did not differ from healthy controls on various executive tasks, such as working memory tests, set shifting, planning or verbal fluency and complex verbal reasoning tasks [19]–[21]. Sustained attentional deficits were reported in drug-naïve patients with NC, but without any relationship to the severity of objective daytime sleepiness [18]. Two other studies stressed poor divided and flexibility performance in patients with NC compared to healthy controls [18], [22]. In sum, the objective evidence for impairment in executive control of attention in NC is contradictory. Furthermore, the performance of patients with NC has never been compared to that of patients with other central hypersomnias in order to determine the respective impact of objective sleepiness and hypocretin deficiency on executive performance.

Theoretical framework of executive control models have stressed the complex relations existing between executive functioning per se and other cognitive areas such as working memory [23] and attention [24]. One comprehensive model identified three independent executive components: (1) inhibition of proponent responses (“Inhibition”), (2) information updating and monitoring (“Updating”), and (3) mental set shifting (“Shifting”) [25].

We hypothesized that executive control of attention problems in narcolepsy would be heterogeneous. One subgroup of patients would have executive performances within normal limits while others would display significant executive impairments. Furthermore, we hypothesized that the severity of objective sleepiness will significantly contribute to the executive performances in patients with narcolepsy, without major differences between patients with and without cataplexy. The aims of this study were (1) to better characterize the executive control of attention in drug-free patients with narcolepsy-cataplexy in comparison to patients with narcolepsy without cataplexy, and to controls matched for sex, age and intellectual level; and (2) to determine the impact of clinical and polysomnographic variables on executive performances in narcolepsy.

## Materials and Methods

### Subjects

Subjects were 22 adult patients with NC (12 males, aged 16–74 years) and 22 adult patients with NwC (14 males, aged 15–65 years) matched for age, gender, intellectual level, objective daytime sleepiness, and number of SOREMPs. Seven patients with NC were treatment-naïve and 15 were drug-free (no psychostimulants or anticataplectic medications) for at least one month prior to evaluation. All patients with NwC were drug-naïve. Narcolepsy diagnosis met the ICSD-2 criteria [26] (The International Classification of Sleep Disorders, 2005): presence of EDS, mean sleep latency of less than 8 minutes, and at least two sleep onset REM periods (SOREMP) during the multiple sleep latency test (MSLT). All patients with NC had clear-cut cataplexy and were HLA DQB1*0602 positive. Patients with NwC had no clinical evidence for cataplexy. HLA DQ typing was performed in 19 patients with NwC with 6 HLA DQB1*0602 positive (30%). No patients had any current psychiatric disorder based on the DSM-IV criteria [27] or associated neurological disorders. Of the 11 patients who had had a lumbar puncture, five patients with NC had undetectable CSF hypocretin-1 levels and six patients with NwC had normal levels (>200 pg/ml).

Thirty-two healthy age-, gender-, and intellectual level-matched controls (15 males, aged 21–53 years) were recruited from the community. All healthy controls were community-dwelling adults living in Montpellier, France, recruited through local associative networks. The inclusion criteria for controls were the ability to understand and give informed consent, no history of neurological or psychiatric disease, and no intake of medications known to influence sleep or cognition. All controls scored <10 on the Epworth Sleepiness Scale (ESS) [28]. HLA typing was not available for controls.

All patients and controls gave their informed written consent to take part in the whole study which was approved by the Local Ethics Committee (University Hospital, Montpellier, France). Additional written consent was obtained from the parents of participants under the age of 18.

### Polysomnography Recordings

All patients (*n = *44) underwent one night of PSG recording followed by the MSLT the next day, consisting of five naps scheduled at 2-hour intervals starting at 9∶00 a.m. [29]. The PSG investigation included measures of sleep, respiratory events, cortical arousals, and periodic leg movements, scored according to standard criteria [30]. Subjects with a respiratory event index (apnea index+hypopnea index) >10 were excluded from the study.

### Clinical and Neuropsychological Evaluation

All participants underwent a standardized face-to-face clinical interview and were asked to complete questionnaires, including the 21-item Beck Depression Inventory-II [31] and the French adaptation of the National Adult Reading Test, fNART [32] to estimate premorbid intelligence quotient (IQ). Patients were all tested after the first MSLT scheduled at 9∶00 AM.

#### Self-Evaluation Attention Questionnaire (QAA) [33]

All subjects completed the QAA. It contains 50 questions divided into seven sections representing different dimensions of concrete activities and situations of daily life: Reading, Conversation, Television, Activities, Distractions, Fatigue, and Driving. Each section includes from 4 to 11 questions. For each question, participants rate on a 6-point scale (from never = 1 to always = 6) the frequency with which they encounter difficulties in a particular situation. A single score per section and a total score are computed to assess the different complaints concerning daily life activities.

#### Executive function assessment

We used a computer-based neuropsychological battery, Zimmermann and Fimm’s attention test battery (Testbatterie zur Aufmerksamkeitsprüfung – TAP) [34], to evaluate attention. This well-validated test battery provides normative data for adults. The tasks consist of simple, easily distinguishable stimuli to which the patient’s motor response is recorded. A button box with millisecond accuracy was used to capture reaction times (RTs) and record responses (false alarms and/or omissions). Based on Miyake and co-workers’ [25] inventory of executive functioning, three subscales of the TAP were used and administered in random order: the 2-Back Task (*Updating*), the Go/No-Go Task (*Inhibition*), and the Flexibility Task (*Shifting*). A fourth task in the TAP, the Tonic/Phasic Alertness Task, was systematically administered at the beginning (Time 1) and end (Time 2) of the evaluation to obtain a behavioural control of fatigability. Each task did not exceed 7 minutes, without any pause proposed in between.

*Tonic and Phasic alertness:* This test was administered in two conditions: tonic and phasic alertness. It was assessed by simple and forewarned reaction time (RT). In phasic condition, the visual target was preceded by a tone. In tonic alertness, participants had to press a button when a visual target (cross) appeared on a screen.

*2-Back Task (Updating):* Participants were presented with a series of digits (ranging from 1 to 9) in a specific sequence. They were instructed to compare each digit with the digit presented two cycles previously and respond by pressing a key.

*Go/No-Go Task (Inhibition):* In this task, a “x” and a “+” were presented. Participants were asked to detect only the cross.

*Flexibility Task (Shifting):* On trial *n*, a letter and a digit were presented on the left or right side of the screen. Participants had to indicate the side where the digit was presented using a right or left button press response. On trial *n*+1, participants were required to detect the side where the letter appeared. Across successive trials, participants had to shift from letter to digit location, and vice versa.

### Statistical Methods

Data were examined for normal distribution and homogeneity of variance. As distributions of QAA measures were skewed, logarithmic transformations were performed using log10X (as recommended by Tabachnik and Fidell), with skewness values being within the acceptable range. For TAP tasks, median RTs, standard deviations (SDs) of RTs, and number of errors (false alarms and omissions) were used as primary measures. PSG and MSLT recording data were scored blinded to the groups and executive performances. Group differences in demographic data, clinical variables, and scale scores were analyzed with one-way between-groups analysis of variance (ANOVA) and Student’s t-tests for independent samples for continuous variables, the Mann-Whitney test for ordinal data, and the χ^2^ test for categorical variables. A 2×2 mixed-factorial repeated measures ANOVA (MANOVA) with group as a between-subjects factor and phasic/tonic alertness tasks (repeated at Time 1 and 2) as a within-subjects factor was performed, using median RTs and SDs for Tonic and Phasic condition as dependent variables. In order to determine executive deficits, data on each executive TAP task were also compared to normative data for RTs, SDs of RTs, and/or errors and omissions, taking into account age and education level [31]. Impairment in executive tasks was defined as a score below the 10th percentile of the norm. The number of impaired executive tasks could therefore vary from 0 to 3. The following variables were considered to determine executive performance: group, estimated intelligence quotient, general slowing at TAP, depressive symptom intensity, objective and subjective sleepiness, SOREMPs at MSLT, sleep efficiency, and SWS and REM percentages. Statistical analyses were performed with SPSS version 12.0 for Windows (Chicago, SPSS, Inc.). The level of significance was α < 0.05.

## Results

### Demographic, clinical, biological, and polysomnographical data

Chi-squared analysis revealed no differences between the two groups of patients and healthy controls in either age or intellectual level. One-way ANOVA indicated a main Group effect for the BDI-II and ESS, with narcoleptic patients reporting higher subjective complaints of sleepiness and higher intensity of depressive symptoms than controls. Differences were also observed in the narcoleptic group with higher BDI-II and ESS scores in patients with NC compared to patients with NwC (respectively, *p* = 0.049 and *p* = 0.039).

**Table 1:** Demographic, clinical, and polysomnographic characteristics of patients with narcolepsy-cataplexy, narcolepsy without cataplexy, and healthy controls.

	Narcolepsywith cataplexy*N* = 22	Narcolepsy without cataplexy *N* = 22	Healthy controls*N* = 32	*p*
*Demographic and clinical data*				
Age	36.2±15.2	29.14±12.5	30.22±8.3	0.19^a^
Men/Women	12/10	14/8	15/17	0.95^b^
Estimated intelligence quotient (IQ)	108.3±6.3	107.0±8.3	110.2±5.1	0.24^a^
Body mass index	24.5±3.9	23.5±4.0	22.7±2.4	0.19^a^
Beck Depression Inventory	16.2±9.7	9.6±10.9	3.9±3.7	<0.001a,d,e
Age at onset, years	22.3±10.3	20.3±9.6	-	0.50^a^
Sleep paralysis,%	68	22	-	0.006^b^
Hypnagogic hallucinations,%	77	22	-	0.001^b^
Epworth Sleepiness Scale	18.5±3.0	16.0±4.3	5.5±2.4	<0.001a,e,f
*Nocturnal sleep parameters*				
Sleep latency	9.5±10.7	31.7±42.1		<0.001^c^
REM latency	30.8±43.2	54.1±31.1		0.03^c^
Total sleep time	428.1±48.9	430.8±66.3		0.87^c^
Sleep efficiency	84.3±8.6	90.4±5.3		0.007^c^
% SWS	19.5±5.4	21.3±6.3		0.31^c^
% REM	23.3±4.7	23.0±6.5		0.86^c^
Apnea/hypopnea index	2.7±3.1	1.6±2.3		0.33^c^
*Diurnal sleep parameters*				
Sleep latency at MSLT†	5.2±1.9	5.9±1.4		0.13^c^
SOREMPs, number†	3.7±1.0	3.3±0.1		0.18^c^

Data are presented as means±standard deviations. PSG refers to Polysomnography; MSLT, Multiple Sleep Latency Test; SOREMPs, Sleep onset Rapid Eye Movement Periods; REM, Rapid Eye Movement; SWS, Slow Wave Sleep. SaO_2:_ Oxygen saturation.

a One-way ANOVA.

b Chi-square test.

c Mann-Whitney test.

d Narcolepsy/Cataplexy vs. Controls.

e Narcolepsy/Cataplexy vs. Narcolepsy without Cataplexy.

f Narcolepsy vs. Controls.

g Narcolepsy without Cataplexy vs. Controls.

Patients with NC had more frequent sleep paralysis (68%) and hypnagogic hallucinations (77%) than patients with NwC (respectively 22% and 22%), with none in controls. No difference was noted between the NC and NwC groups in age at disease onset. The NC and NwC groups were matched for mean sleep latency and number of SOREMPs on the MSLT. Patients with NC had lower sleep efficiency, sleep and REM sleep latencies than patients with NwC. They also had shorter REM sleep latency (Table 1).

### Self-Evaluation Attention Questionnaire

**Figure 1:**
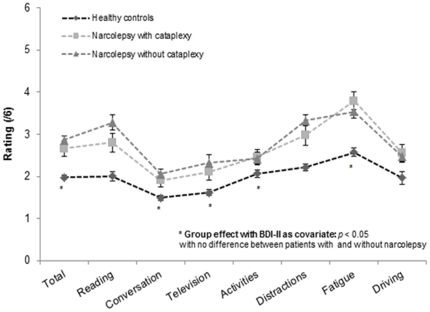
Score obtained on the 7 sections of the Self-Evaluation Attention Questionnaire (QAA) for patients with narcolepsy and for healthy controls. Mean (±SEM) are given.

Significant correlations were found between sections of the QAA and the BDI-II scale in the control group and in patients with narcolepsy. Group comparison analyses on the QAA were thus performed with the BDI-II score as covariate. Figure 1 displays the scores obtained by patients with narcolepsy (with and without cataplexy, N = 44) and healthy controls (N = 32) on each section of the QAA and total scores. Patients with NC and NwC reported higher attentional complaints than controls excepted for Reading, Distractions and Driving sections. Bonferroni post hoc tests indicated no significant difference between patients with NC and NwC (all *p-values*>0.20). We found no association between the attentional complaint level, demographical, clinical and polysomnographical variables.

### Executive Functions Assessment

#### Group comparisons: Tonic and Phasic alertness

**Figure 2:**
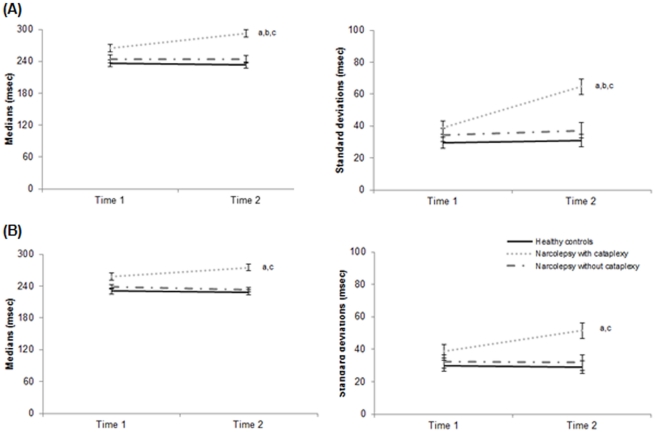
TAP median (left panel) and standard deviation (right panel) reaction times across Tonic (A) and Phasic (B) Task for patients with narcolepsy and for healthy controls. The Tonic/Phasic Task was systematically administered at the beginning (Time 1) and end (Time 2) of the evaluation. Means (±SEM) are given. [Group^a^, Time^b^, Group x Time^c^ effects: all *p-value*s<0.001]

The analysis of Tonic alertness median and SDs of RTs showed a significant main effect of Group [respectively: *F*(2,72) = 14.4, *p*<0.001, *η* ^2^ = 0.18; *F*(2,72) = 11.7, *p<*0.001, *η* ^2^ = 0.24] and Time [respectively: *F*(2,72) = 5.3, *p* = 0.024, *η* ^2^ = 0.06; *F*(2,72) = 10.6, *p* = 0.002, *η* ^2^ = 0.12], and a significant Group x Time interaction [respectively: *F*(2,72) = 8.05, *p* = 0.001; *F*(2,72) = 6.4, *p* = 0.003, *η* ^2^ = 0.15] (Figure 2A). Post hoc multiple comparisons with Bonferroni corrections indicated that patients with NC showed significantly slower RTs and more variable SDs of RTs compared to healthy controls (all *p-values*<0.001) and patients with NwC (all *p-value*s<0.01), whereas patients with NwC did not differ from controls (all *p-value*s >0.50). In addition, patients with NC were more affected by testing session than patients with NwC (all *p-value*s<0.001) and controls (all *p-value*s<0.001), with slower and more variable RTs at Time 2. The same results pattern was observed for Phasic alertness median and SDs of RTs, with significant effects of Group and Time and a significant Group x Time interaction (Figure 2B). We may add that none of participants fell asleep during the testing session.

#### Updating, inhibition, and flexibility

**Figure 3:**
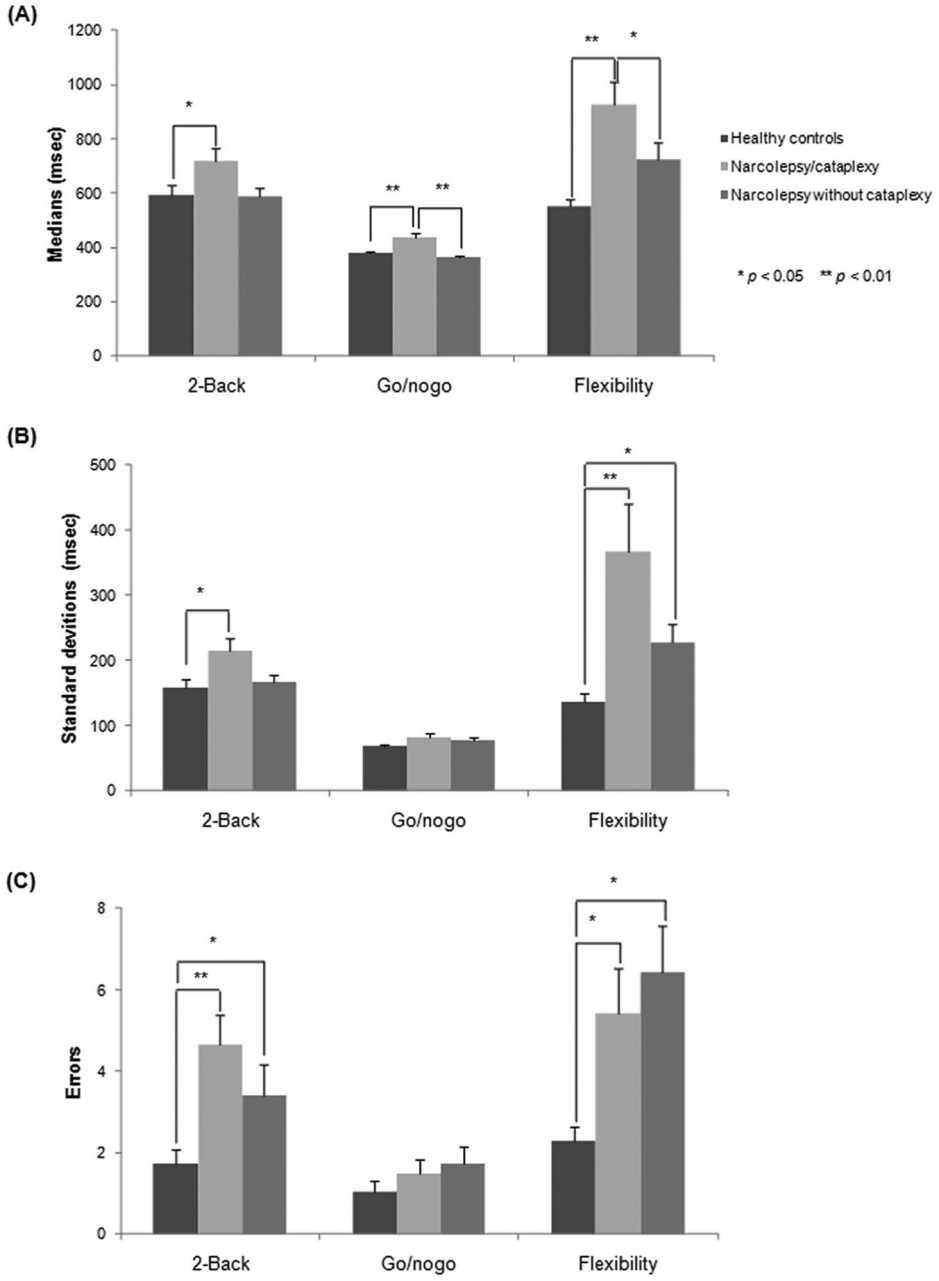
TAP median reaction times (A), standard deviation reaction times (B) and errors (C) for patients with narcolepsy and for healthy controls. Means (± SEM) are given.

An ANOVA performed on median of RTs indicated a main Group effect for all executive tasks [2-Back: *F*(2,72) = 3.4, *p* = 0.038, *η* ^2^ = 0.08; Go/No-Go: *F*(2,72) = 9.9, *p*<0.001, *η* ^2^ = 0.23; Flexibility: *F*(2,72) = 13.3, *p* = 0.001, *η* ^2^ = 0.27] (Figure 3A). Post hoc analyses revealed that patients with NC were slower than healthy controls on all executive tasks (all *p-values* <0.05) and slower than patients with NwC on the Go/No-Go (*p*<0.001) and Flexibility (*p* = 0.046) tasks. The ANOVA conducted on SDs of RTs showed a Group effect for the 2-Back [*F*(2,72) = 4.3, *p* = 0.016, *η* ^2^ = 0.11] and Flexibility [*F*(2,72) = 8.1, *p* = 0.001, *η* ^2^ = 0.18] tasks (Figure 3B). Contrast analyses showed that patients with NC had more variability in performance than controls on the 2-Back (P = 0.017) and Flexibility (*p*<0.001) tasks. Patients with NwC showed more variable performance than controls on the Flexibility task (*p* = 0.042). Kruskal Wallis tests performed on error rates revealed a main Group effect for the 2-Back (*Z* = 12.1, *p* = 0.002) and Flexibility (*Z* = 11.3, *p* = 0.003) tasks (Figure 3C). Contrast analyses showed that patients with NC were less accurate than controls (2-Back, *p* = 0.01; Flexibility, *p* = 0.048).Patients with NwC made also more errors than controls (2-Back, *p* = 0.024; Flexibility, *p* = 0.001). No significant associations were found between BDI-II, ESS scores, Tonic and Phasic alertness and complex executive performances in the whole sample and in patients with narcolepsy only.

### Individual Performance Patterns

Patients and controls were compared to the normative data on the four TAP tasks, taking into account age and education level [34].

#### Tonic and Phasic alertness

Most of patients showed normal performance at Time 1, but not at Time 2 with both Phasic (*n* = 11) and Tonic (n = 10) alertness impairment. In contrast, two controls only had abnormal performance on Phasic (*n* = 1) and Tonic (n = 1) alertness. Contrast analyses indicated that patients with NC were slower than patients with NwC on the Phasic (*χ* ^2^ = 9.2, *p* = 0.002) and Tonic (*χ* ^2^ = 5.6, *p* = 0.018) alertness tasks. Patients with the most Phasic and Tonic alertness impairment at Time 2 had the shortest MSLT latency (respectively, *Z* = −2.08, *p* = 0.036; *Z* = −1.8, *p* = 0.05).

#### Updating, inhibition, and flexibility

**Figure 4:**
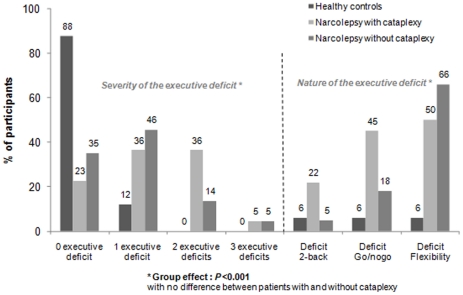
Rate of participants showing impairments in none, 1, 2, and 3 executive TAP tasks and in the nature of the executive deficit.

The number of altered executive tasks (updating, inhibition, and flexibility) differed between patients with NC, NwC, and controls (χ^2^ = 37.0, *p*<0.001) (Figure 4). Our controls performed normally on 88% of the TAP tasks, in contrast to patients with NC (23%) and NwC (35%). However, no between patient group differences were noted on the number and type of impaired executive tasks (Figure 4).

### Analyses of Correlates

To study the correlates of the deficit in complex executive performances, patients with narcolepsy (*N* = 44) were split into two groups, those with at least one executive deficit (n = 32) and those without (*n* = 12). Patients with altered performance had shorter MSLT latency (*Z* = −2.71, *p* = 0.006), higher SOREMPs (*Z* = −2.40, *p* = 0.019), and lower estimated intellectual level (*Z* = −2.28, *p* = 0.021) than patients without. The number of SOREMPs was negatively correlated with MSLT latencies (*r* = −0.32; *p* = 0.01) in patients with narcolepsy. Interestingly, no between-group differences on executive deficit exist regarding the Phasic/Tonic Alertness tasks at Times 1 and 2, and the QAA evaluation.

## Discussion

This study was the first to investigate the executive control of attention in a sizeable group of drug-free patients with NC compared to patients with NwC and healthy controls. The main question of the present study was whether the observed executive deficits in narcolepsy are specific to its pathophysiology or whether they reflect performance changes due to the severity of objective daytime sleepiness. By comparing patients with NC and NwC matched for MSLT latencies, we aimed to control for the influence of sleepiness on executive performance in the patient groups.

NC is characterized by loss of hypocretin-containing neurons with widespread projections throughout the brain, including regions involved in the executive network [35], [36]. Recent studies have reported that the hypocretin system increases emotion-related behavioural responses [14]. In contrast, the large majority of patients with NwC (70–90%) had normal CSF hypocretin1-levels [4], [9], [37], [38]. We found in the present study that, compared to controls, patients with narcolepsy reported higher attentional complaints, without any difference between patients with NC and NwC. Patients with NC were slower and more variable on simple reaction time tasks, especially when performed the second time, compared to patients with NwC, without between NwC and control differences. Regarding the executive functioning, patients with narcolepsy generally performed slower, reacted more variably, and made more errors than controls, showing a clear heterogeneity of the severity of updating, inhibition, and flexibility performances. The nature and severity of the executive deficits were unrelated to the diagnosis of NC and NwC, and therefore unrelated to hypocretin deficiency *per se*, but rather to objective sleepiness, higher number of SOREMPs, and lower intellectual quotient. Altogether, our results suggest that patients with NC with hypocretin deficiency had low performances on both alertness and complex executive tasks. In contrast, the unyet-established neurobiological defect in NwC appears unable to induce a general cognitive slowing on simple reaction time but may alter performances on tasks requiring higher cognitive demand.

Few studies have addressed objective cognitive changes in association with narcolepsy, and the results are contradictory [18]–[22]. Several methodological aspects specifically related to the executive functioning evaluation could explain these discrepancies. The first is that a single task may be inadequate to capture complex executive disturbances [12]. The second is related to the overlapping between measures of intellectual level and executive functioning [39]. Third, although some studies have reported anatomic differences in attentional networks for vigilance and executive functions, there is clear evidence that these networks interact [13]. Finally, the few studies that have addressed executive changes in association with narcolepsy included small clinical samples of patients with mixed conditions (narcolepsy with and without cataplexy, and both drug-naïve and medicated patients), such that cognitive problems may have been underestimated [18], [22], [40].

This study reported for the first time an individual approach to the executive control of attention in narcolepsy. In relation to the idea of the executive control of attention as a non-unitary concept, we decided to assess executive functioning in patients and controls using a fractionated approach based on a seminal cognitive study [25].

We noted that estimated intellectual level was related to the severity of executive TAP task impairments in patients with NC and NwC. Although accurate analysis of executive performance requires knowledge of individual performance on intelligence measures, most cognitive studies in narcolepsy have not controlled for this variable [19], [22]. Performance on executive tasks in patients with central hypersomnias requires careful analysis and interpretation [41]. Alertness refers to the intrinsic arousal that fluctuates on the order of minutes to hours, being intimately involved in sustaining attention but also in the cognitive tone required to perform the complex executive control [42], [43]. Hence, experimental sleep deprivation studies in healthy controls had wide ranging effects on brain functioning, affecting multiple, distinct components of cognition but especially the cognitive processes mediated by the prefrontal cortex, thereby degrading both attentional arousal and higher-order cognitive functions, such as executive control of attention [44], [45]. As patients with central hypersomnia had reduced arousal and vigilance in executive processes, the effects of alertness/sleepiness should always be controlled for in assessing the executive control of attention.

Two limitations in our study need to be addressed. First, CSF hypocretin-1 level was determined in only five patients with NC and six patients with NwC. In future, it would be useful to relate the CSF hypocretin-1 level of patients with narcolepsy (with and without cataplexy) to their executive performance. Secondly, the sample size is relatively low (22 patients with NC and 22 with NwC compared to 32 age-, gender-, and intellectual level-matched controls), although sufficient to demonstrate significant reduced performance on executive control attention tasks.

In sum, we demonstrated that drug-free patients with NC had low performances on both alertness and executive tasks, in contrast to patients with NwC with altered results only on tasks requiring higher cognitive demand. The altered executive control of attention is clearly heterogeneous in narcolepsy, being somehow independent of the hypocretin deficiency per se, but mostly explained by the severity of objective sleepiness and global intellectual level. Further studies are needed to explore whether medications that promote wakefulness can improve the executive functions in narcolepsy.
